# Delayed Oral LY333013 Rescues Mice from Highly Neurotoxic, Lethal Doses of Papuan Taipan (*Oxyuranus scutellatus*) Venom

**DOI:** 10.3390/toxins10100380

**Published:** 2018-09-20

**Authors:** Matthew R. Lewin, José María Gutiérrez, Stephen P. Samuel, María Herrera, Wendy Bryan-Quirós, Bruno Lomonte, Philip E. Bickler, Tommaso C. Bulfone, David J. Williams

**Affiliations:** 1 Ophirex, Inc., Corte Madera, CA 94925, USA; tommaso.bulfone@gmail.com; 2 California Academy of Sciences, San Francisco, CA 94118, USA; paulshania@yahoo.co.uk; 3 Facultad de Microbiología, Instituto Clodomiro Picado, Universidad de Costa Rica, an José 11501-2060, Costa Rica; maria.herrera_v@ucr.ac.cr (M.H.); wenjbq@gmail.com (W.B.-Q.); bruno.lomonte@ucr.ac.cr (B.L.); 4 Queen Elizabeth Hospital, Kings Lynn, Norfolk PE30 4ET, UK; 5 Anesthesia and Perioperative Care, University of California San Francisco, San Francisco, CA 94143, USA; philip.bickler@ucsf.edu; 6 Department of Pharmacology and Therapeutics, Australian Venom Research Unit, University of Melbourne, Parkville, VIC 3010, Australia; david.williams@unimelb.edu.au

**Keywords:** snakebite, envenoming, neglected tropical disease, field antidote, inhibitor, taipan, PLA2, phospholipase A2, neurotoxicity, antivenom

## Abstract

There is an unmet need for economical snakebite therapies with long shelf lives that are effective even with delays in treatment. The orally bioavailable, heat-stable, secretory phospholipase A_2_ (sPLA_2_) inhibitor, LY333013, demonstrates antidotal characteristics for severe snakebite envenoming in both field and hospital use. A murine model of lethal envenoming by a Papuan taipan (*Oxyuranus scutellatus*) demonstrates that LY333013, even with delayed oral administration, improves the chances of survival. Furthermore, LY333013 improves the performance of antivenom even after it no longer reverses neurotoxic signs. Our study is the first demonstration that neurotoxicity from presynaptic venom sPLA_2S_ can be treated successfully, even after the window of therapeutic antivenom has closed. These results suggest that sPLA_2_ inhibitors have the potential to reduce death and disability and should be considered for the initial and adjunct treatment of snakebite envenoming. The scope and capacity of the sPLA2 inhibitors ability to achieve these endpoints requires further investigation and development efforts.

## 1. Introduction

Snakebite envenoming is a threat to more than five billion people and at least 750 million who live more than 1 h from a health care facility [1]. Additionally, snakebite envenoming also represents a substantial risk to livestock [2]. In the modern era, snakebite envenoming kills 81,000–138,000 people annually and leaves hundreds of thousands with amputations, disfiguring injuries, and both chronic physical and psychological sequelae [3,4]. Nearly 130 years ago, Sir Joseph Fayrer wrote, “Until some such measures are generally and systematically resorted to, there will be no diminution in loss of human life from snakebite” [5]*.* Only in the last two years has snakebite received recognition as a Neglected Tropical Disease; Fayrer′s words are as relevant today as they were more than a century ago [3,5].

Currently, the definitive treatment for snakebite envenoming requires the intravenous administration of animal-derived antivenoms at a health care facility [3,6]. Use of intensive and often catastrophically expensive resuscitative measures such as mechanical ventilation and operative interventions have reduced the rates of in-hospital morbidity and mortality from snakebite. However, more than 75% of deaths from snakebite envenoming occur before reaching hospital care [1,7]. Poverty and proximity to hospital care are risk factors associated with poor outcomes [1,3,8]. An effective, field-administered antidote to snakebites would address many of the limitations of antivenom and reduce both the economic and technical barriers to initiating care for these life and limb threatening emergencies [9,10,11,12,13,14,15,16].

Snake venom phospholipases (PLA_2S_) are almost universally present amongst the world’s most lethal venomous snakes [17]. The blockade of these enzymes after an envenoming snakebite could prevent rapid lethality from neurotoxicity, as well as potentially reduce other harmful morbidities, such as myonecrosis, acute kidney injury, and venom-induced endogenous inflammatory responses [18,19,20,21,22].

LY315920 and its orally bioavailable prodrug, LY333013, have been found to have extremely potent, broad-spectrum activity against snake venom PLA_2S_ and have been proposed as a promising initial treatment for envenoming snakebite [9]. This work was recently replicated and extended when LY315920 preserved life and prevented haemorrhagic, myonecrotic, and neurological effects in mice injected with krait, cobra, or viper venoms from medically important species in China [23]. LY315920 and LY333013 were previously abandoned following Phase II and Phase III of human clinical trials for sepsis, rheumatoid arthritis, and cardiovascular disease, but could be repositioned at a low development cost for the initial treatment of snakebite [24]. Along with known safety profiles, LY315920 and LY333013 pharmacodynamics and pharmacokinetics in short-term use is well-understood, but unfortunately these potent sPLA2-blocking drugs were abandoned for lack of efficacy after more than 25 clinical trials; they were never considered for use in the setting of snakebite [24]. As proof of a concept experiment, we sought to examine how these drugs might perform both alone and with antivenom in a scenario of lethal envenoming by Papuan taipan. Papuan taipan venom was selected because its main toxicity relies on the action of a potent presynaptically-acting neurotoxic heterotrimeric PLA_2_, taipoxin [18,25].

The key unknown in this experiment was whether LY333013 would be effective in preventing neurotoxicity and lethality from Papuan taipan *(Oxyuranus scutellatus)* venom if given orally after venom injection, both immediately after envenoming and at a time when specific antivenom is no longer effective in preventing lethality. To begin answering this question, we tested the ability of LY333013 (10 mg/kg) when administered orally. We administered LY333013 either immediately (within five min) or in one h (60 min) following the subcutaneous administration of venom. We used an empirically selected venom dose ultimately corresponding to 12LD_50S_, in order to model a severe envenoming. Mice died within a range of three to five h, thus allowing for the administration of the drug after envenoming. The LY333013 dose was chosen based on historical preclinical and human clinical trial data at a dose deemed well within the known safety margin [24]. The effect of the drug, when administered orally, was compared to a monospecific taipan antivenom, administered by the intravenous route at a dose of 5 mL/kg in fewer than five min (“urgent” administration) or at 60 min following administration of the venom (“delayed” administration). Additionally, to examine another clinically plausible scenario, antivenom was administered one h after envenoming, immediately followed by oral administration of LY333013, as it might be administered in the acute setting such as entry to the hospital triage, but prior to antivenom administration. A detailed description of the procedures is included in the Methods section.

## 2. Results

**Figure 1:**
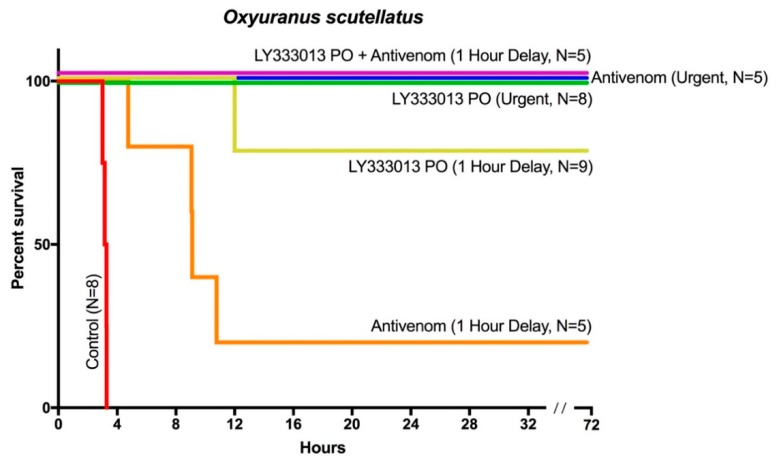
A single oral dose of LY333013 restores the effect of antivenom even after an otherwise fatal delay in antivenom administration. A single oral dose of LY333013 (10 mg/kg) or intravenous taipan specific antivenom (5 mL/kg) administered urgently (within 5 min) following subcutaneous injection of 12 × LD_50_ *O. scutellatus* venom resulted in survival of all tested mice. A single dose of oral LY333013 administered with a 60 min delay following the 12 × LD_50_ resulted in survival of seven out of nine mice, whereas only one out of five mice receiving antivenom with a 60 min delay survived. The combination of LY333013 and antivenom at this same 60 min time resulted in survival for all mice.

Mice to whom 12LD_50_ doses of venom were given with no treatment died within three h. In contrast, mice receiving either LY333013 or antivenom less than five min after venom injection showed no neurotoxic manifestations, e.g., limb paralysis or labored breathing. Mice treated urgently with LY333013 survived the 72 h observation period without sequelae (Figure 1). When LY333013 dosing was delayed for one h, seven out of nine envenomed mice treated with LY333013 survived without further intervention, whereas only one out of five mice receiving antivenom after one h of envenoming survived. However, when both LY333013 and antivenom were administered one h after venom injection, all mice survived without residual signs of neurotoxicity (Figure 1). Thus, the concomitant administration of antivenom and LY333013 appears to be more effective than either treatment alone. We predict that the dual use of inhibitor-antivenom combinations is a likely clinical scenario in the evolution of snakebite treatments.

## 3. Discussion

The effectiveness of antivenom to prevent the development of neurotoxicity in mice injected with taipan venom correlates with clinical observations indicating that patients receiving antivenom within four h (generally prior to onset of severe neurotoxicity) are significantly less likely to develop life-threatening oropharyngeal neuromuscular paralysis. By contrast, >60% of patients in whom antivenom administration was delayed >4 h required endotracheal intubation and mechanical ventilation, often for prolonged periods, in order to sustain life [26]. In our mouse model, the limitations of antivenom administered one h after envenoming is in agreement with clinical reports of progressing neurotoxicity despite antivenom in patients who have already developed signs of neurotoxicity prior to antivenom administration [26,27]. This underscores one of the limitations of antivenom therapy, especially in rural settings where the arrival to medical facilities may take several hours. Furthermore, the limitations of antivenom additionally highlights the relevance of developing inexpensive therapies that could be used immediately following the bite of a snake safely in the pre-hospital setting, with or without specific species identification or any special training.

Our observations of LY333013 are important, since oral administration of this drug prevented both the development of neurotoxicity when given early, and also prevented neurotoxicity and lethality past the point at which antivenom could treat severe envenoming. To our knowledge, our study is the first demonstration that shows an intervention outside the range at which antivenom can prevent neurotoxicity, other than sustained mechanical ventilation to rescue animals from lethal envenoming [28]. The mechanisms behind this phenomenon need to be further investigated. Three substituted indoles from which LY333013 is a member could be an invaluable research tool in understanding fundamental aspects of sPLA_2_ in the context of snakebite envenoming.

The combination of LY333013 and antivenom, when treatment was delayed for one h, gave better results than either treatment alone. Since taipan venom has other toxic components in addition to taipoxin, such as α-neurotoxins and procoagulant serine proteinases [29], the neutralization of these toxins by antivenom antibodies complements the inhibition of taipoxin by antibodies and LY333013, when in circulation or when LY333013 has reached peripheral tissues. Thus, our results suggest that early oral administration of this PLA_2_ inhibitor in the field or hospital, followed by the infusion of antivenom at a medical facility could be a robust therapeutic approach for envenomings by Papuan taipans and, potentially, other types of snakebites whose main toxicity is based on the action of PLA_2S_ or a severe inflammatory response to other venom components mediated by endogenous sPLA_2_ [3,22,30].

Oral LY333013 and its intravenously administered active ingredient, LY315920, are small molecules ripe for further testing in the pipeline of drug candidates being considered for development as initial treatments and antivenom adjuncts [9,23]. In the situation of a lethal taipan bite, the preclinical efficacy, safety profile of three substituted indoles, and availability of high quality antivenom and hospital care, suggest the need for trials of drug-antivenom combinations. Further examination of these inhibitors for other snake venom-induced _S_PLA2 pathology is additionally warranted [19,23,30].

## 4. Materials and Methods

### 4.1. Experimental Design to Assess the Efficacy of Drug and Antivenom

Groups of CD-1 mice of both sexes (18–20 g body weight) were used. We utilized Prism 7 (GraphPad Software, Inc., La Jolla, CA, USA) for the statistical analyses based on historical data from similar experiments we had performed on CD-1 mice. The experimental design was approved by the Institutional Committee for the Care and Use of Animals (CICUA) of the University of Costa Rica (CICUA 27-14, 15 July 2014) and met the International Guiding Principles for Biomedical Research Involving Animals (CIOMS).

Mice were subcutaneously injected with 100 µL of PBS containing 3 µg venom. This corresponds to approximately 12 Median Lethal Doses (LD_50_) of venom. The dosage was chosen based on time to lethality deemed appropriate for a model of severe envenomation and therapeutic intervention [31]. A control group of mice received an oral dose of 200 µL of gum Arabic solution (8% *w/v*) after venom injection. Two groups of envenomed mice were treated immediately after venom injection with either LY333013 (a dose of 10 mg/kg administered orally in 200 µL gum Arabic solution) or with antivenom (a dose of 5 mL/kg by the intravenous route in a volume of 100 µL). Two additional groups of envenomed mice were treated with the same doses of LY333013 and/or antivenom one h after venom injection. Mice were observed continuously for at least the first 12 h and deaths recorded. Deaths occurring during the 72 h experimental period were recorded as the time last observed, regardless of clinical condition.

### 4.2. Venom

The venom of adult specimens of *Oxyuranus scutellatus* from Papua New Guinea was kindly provided by the University of Melbourne. Upon extraction, venom was snap frozen and then freeze-dried. Venom solutions were prepared immediately before use by dissolving dry venom in 0.12 M NaCl, 0.04 M phosphate, pH 7.2 (PBS). Subcutaneous LD_50_ for this specific batch of venom was previously established by Herrera et al [31] and is the same batch used to produce taipan specific antivenom for Papua New Guinea by the manufacturer, Instituto Clodomiro Picado.

### 4.3. sPLA_2_ Inhibitor and Antivenom

The PLA_2_ inhibitor LY333013 (CAS NO: 172733-08-3), with a molecular weight of 394.4, was supplied by Ophirex, Inc. (Chemietek, Indianapolis, Indiana; Lot 02: 99.99% purity by NMR). LY333013 was then dissolved in 8% *w/v* gum Arabic in water based on empirical experience related to reliability of mixing, as well as ease of passage through appropriately sized stainless-steel rodent feeding needles. The monospecific taipan antivenom produced at Instituto Clodomiro Picado (University of Costa Rica; batch number 4511209) was used. It was a whole IgG preparation obtained by caprylic acid precipitation of plasma of horses immunized with the venom of *O. scutellatus* from Papua New Guinea. The Median Effective Dose (ED_50_) of the antivenom, when tested following World Health Organization standard procedures—i.e., incubating venom and antivenom before injection in mice is 3.96 mg venom neutralized per mL antivenom (95% confidence limits: 2.99–5.54 mg venom/mL antivenom).
